# The effect of green exercise on mobile phone addiction among university students: a chain mediating role of perceived social support and negative emotions

**DOI:** 10.3389/fpsyg.2026.1876698

**Published:** 2026-08-11

**Authors:** Ke Shen, Yue Li, Jiaxing Tang, Gaopeng Chen

**Affiliations:** 1 School of Physical Education and Health, Jiangsu University of Architecture Technology, Xuzhou, China; 2 School of Physical Education (School of Soccer), Huaibei Normal University, Huaibei, China; 3 School of Physical Education, Xiangnan University, Chenzhou, China

**Keywords:** college students, green exercise, negative emotions, perceived social support, smart phone addiction

## Abstract

**Objective:**

This study aimed to investigate the correlational mechanisms linking green exercise, perceived social support, negative emotions, and mobile phone addiction among university students.

**Methods:**

Using a stratified cluster sampling method, a total of 1,000 university students (aged 19.15 ± 1.08 years) were recruited from six universities randomly selected across Anhui, Shandong, and Jiangsu provinces. Participants completed Intentional Nature Exposure Scale (INES), the Perceived Social Support Scale, the Depression Anxiety Stress Scale (DASS-21), and the Smartphone Addiction Scale for College Students. Data were analyzed using SPSS 23.0 and Hayes’ PROCESS macro. Reliability and validity analyses, correlation analysis, regression analysis, structural equation modeling, and bias-corrected percentile bootstrap tests were sequentially conducted on the study variables.

**Results:**

(1) Green exercise was significantly and negatively associated with mobile phone addiction (*β* = −0.214, *p* < 0.01). (2) Green exercise was significantly and positively associated with perceived social support (*β* = 0.645, *p* < 0.01) and significantly and negatively associated with negative emotions (*β* = −0.326, *p* < 0.01). (3) Perceived social support was significantly and negatively associated with both negative emotions (*β* = −0.314, *p* < 0.01) and mobile phone addiction (*β* = −0.144, *p* < 0.01). (4) Negative emotions were significantly and positively associated with mobile phone addiction (*β* = 0.278, *p* < 0.01).

**Conclusion:**

Perceived social support and negative emotions serve as mediators in the relationship between green exercise and mobile phone addiction, with a total indirect effect estimate of −0.240. This study indicates that green exercise is not only directly associated with mobile phone addiction but is also indirectly associated with it through the chain mediating effects of perceived social support and negative emotions.

## 1 Introduction

Smartphones, as indispensable tools in modern society, facilitate daily life while simultaneously posing a risk of excessive dependence and even addiction. Mobile phone addiction refers to an addictive behavior characterized by the uncontrolled and excessive use of mobile phones, leading to impaired social functioning and subsequent psychological and behavioral problems (Liu et al., 2017). University students represent a high-risk population for mobile phone addiction, with surveys indicating that over 36% exhibit addictive behaviors (Peng et al., 2024). This condition not only results in deteriorated vision, poor sleep quality, and declining academic performance (Dridi et al., 2025) but is also highly correlated with psychological issues such as depression, stress, and anxiety (Li et al., 2020; Zhang et al., 2022). Therefore, identifying effective strategies to prevent and mitigate mobile phone addiction is significant for the physical and mental well-being of university students.

While traditional interventions, such as cognitive-behavioral therapy and time management training, have demonstrated efficacy, they are often limited by high implementation costs and low student engagement. In recent years, green exercise has garnered widespread attention due to its dual advantages of environmental restoration and physical activity promotion. Green exercise is defined as physical activity undertaken in natural environments, integrating both contact with nature and bodily movement. Research has shown that green exercise can reduce levels of the stress hormone cortisol and enhance positive emotions (Lahart et al., 2019). However the mechanisms through which it relates to mobile phone addiction remain poorly understood.

Grounded in ecological systems theory and a positive psychology perspective, the present study aims to elucidate the mechanisms underlying the effect of green exercise on mobile phone addiction among university students. The findings are intended to provide a practical basis for cultivating healthy smartphone usage habits and improving the physical and mental health of university students, as well as to offer theoretical guidance for the reform of physical activity programs in higher education and the planning of spaces for physical activity on campus.

## 2 Theoretical background

### 2.1 Green exercise and mobile phone addiction

According to ecological systems theory, the natural environment constitutes one of the microsystem factors influencing individual behavior. Exposure to green environments has been shown to improve individuals’ mood states, reduce stress, and enhance well-being. Physical activity performed within such green environments appears to yield similar, if not superior, benefits. Green exercise is defined as physical activity undertaken in natural environments, particularly those characterized by green spaces (Pretty et al., 2005). Research indicates that engaging in outdoor exercise in natural or man-made green environments, performing indoor exercise while viewing natural surroundings or images of natural landscapes through a window, or even experiencing virtual natural environments can all improve positive mood levels, with effects superior to those observed in non-green environments (Gao et al., 2023). Mobile phone addiction is characterized by excessive smartphone use that leads to physical, psychological, and social functional impairments, manifesting as cognitive dysfunction and behavioral abnormalities (Su et al., 2025). Studies have demonstrated a significant negative correlation between the severity of mobile phone addiction and physical activity levels among university students (Han et al., 2023), Furthermore, mobile phone addiction has been found to be significantly and negatively associated with physical activity levels (Feng et al., 2022). Currently, most research on green exercise has focused on its effects on mood improvement, stress reduction, and sleep quality enhancement; however, its impact on mobile phone addiction remains unclear. Based on the aforementioned theoretical and empirical evidence, the present study proposes the following hypothesis H1: Green exercise is negatively associated with mobile phone addiction among university students.

### 2.2 The mediating role of perceived social support

Self-determination theory posits that humans have three fundamental psychological needs: competence, relatedness, and autonomy. Among these, the need for relatedness reflects the requirement for social support, which is considered an essential element for the healthy development of an individual’s physical and psychological well-being. Perceived social support refers to an individual’s subjective experience of feeling respected, supported, and understood within their social, family, and school contexts, as well as their level of satisfaction with this support (Ding et al., 2025). For university students experiencing mobile phone addiction, objective social support is often severely diminished, leading to a corresponding reduction in perceived social support and consequently generating a strong yearning for social connection.

The social buffering theory posits that robust social relationships can provide emotional comfort and practical assistance, thereby reducing an individual’s reliance on virtual worlds (Bai et al., 2025). Green exercise, often undertaken in group formats such as outdoor hiking or fitness activities, can increase opportunities for interpersonal interaction among university students, strengthen social relationships, and facilitate the acquisition of social support. Research has found that university students who participate in green exercise are more likely to receive support from parents and peers, enhancing their sense of social belonging and subsequently reducing mobile phone addiction behaviors driven by loneliness (Bai, 2022). Based on the theoretical framework and empirical evidence outlined above, the present study proposes the following hypothesis H2: Perceived social support mediates the relationship between green exercise and mobile phone addiction among university students.

### 2.3 The mediating role of negative emotions

Smartphones, with their multifunctional capabilities encompassing social interaction, entertainment, and shopping, serve as the most convenient and accessible communication tools in contemporary society. Consequently, they readily become a means for individuals to alleviate stress or cope with social anxiety. Research indicates that the entertaining and novel content delivered via smartphones can rapidly divert attention, temporarily disengaging the brain from real-world scenarios that generate negative emotions and facilitating a brief emotional detachment. This short-term emotional relief can foster a strong psychological dependence, ultimately evolving into addictive behavior (Zhang et al., 2017). Conversely, negative emotions can diminish an individual’s resistance to addictive substances or behaviors by impairing self-control and inhibiting executive functions (Zhou et al., 2017; Tong et al., 2019), thereby contributing to mobile phone addiction. Green exercise, by contrast, promotes the release of endorphins and serotonin while reducing cortisol levels. This physiological relaxation helps alleviate tension and mitigate negative emotions at a biological level, which in turn may reduce mobile phone addiction behaviors driven by such emotional states.

Based on the theoretical reasoning and empirical evidence presented above, the present study proposes the following hypothesis H3: Negative emotions mediate the relationship between green exercise and mobile phone addiction among university students.

### 2.4 The chain mediating role of perceived social support and negative emotions

Group interaction serves as a crucial social vehicle for green exercise. Through collaborative physical activities and mutual assistance in outdoor natural settings, university students are more likely to perceive emotional care and behavioral support from their peers. This, in turn, strengthens their subjective recognition of the surrounding social support network, thereby enhancing their level of perceived social support. When individuals subjectively perceive stable social support, they further acquire emotional affirmation and a sense of connection within the group, fostering a strong sense of psychological belonging (Luo et al., 2025), This sense of belonging effectively satisfies university students’ social and emotional needs, consequently attenuating the emergence and persistence of negative emotions such as loneliness, anxiety, and depression. Negative emotions are closely associated with mobile phone addiction (Wang et al., 2026). A reduction in the level of negative emotions can directly diminish individuals’ behavioral motivation to seek immediate emotional comfort or escape reality through their smartphones. This disruption of the conditioned reflex wherein negative emotions lead to excessive smartphone use ultimately contributes to the effective inhibition of mobile phone addiction behaviors among university students. Based on the theoretical reasoning outlined above, the present study proposes the following hypothesis H4: Green exercise is associated with mobile phone addiction among university students through the chain mediating effects of perceived social support and negative emotions.

**Figure 1:**
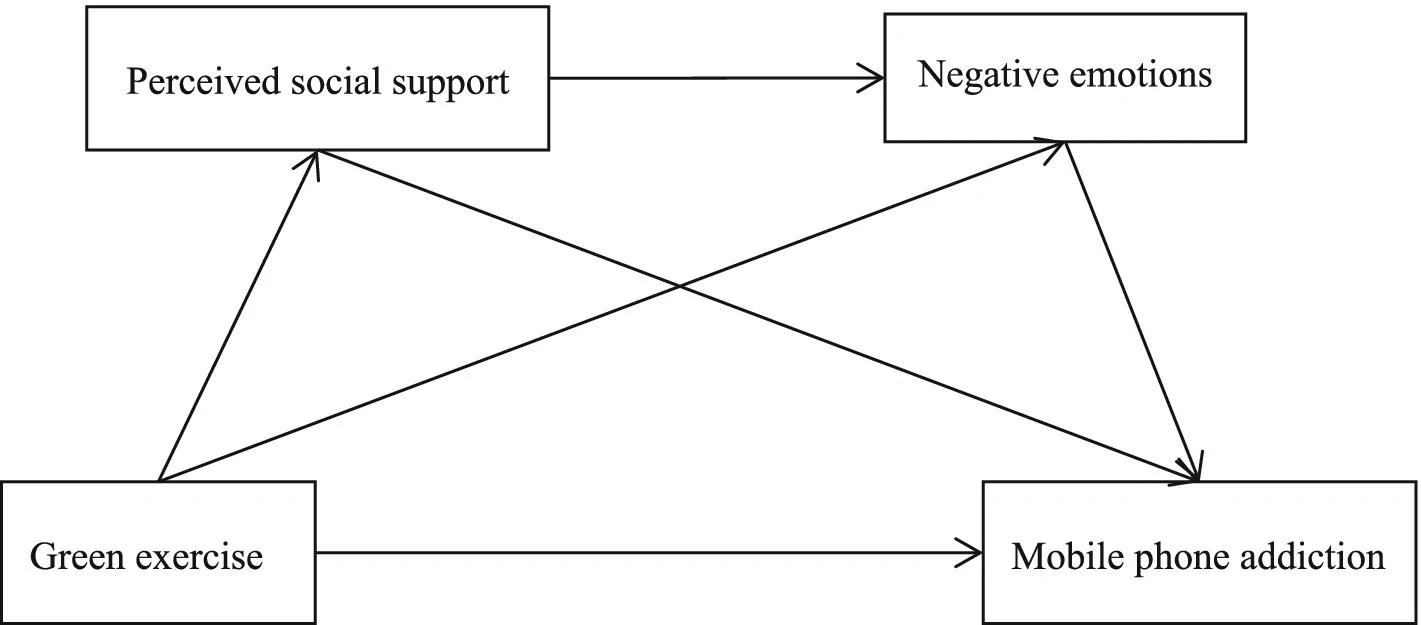
Conceptual framework.

In accordance with the hypotheses formulated above, this study constructed a chain mediation model (Figure 1). The model aims to provide insights for reducing mobile phone addiction behaviors among university students, promoting the development of healthy physical exercise habits, and offering theoretical references for relevant stakeholders in policy formulation and decision-making.

## 3 Methods

### 3.1 Participants

Using a stratified cluster sampling method, two junior colleges, two general undergraduate universities and two key universities were chosen from Anhui, Shandong and Jiangsu Provinces respectively, and 1,000 university students were randomly selected as samples. Sampling procedures ensured the representativeness of institution types and academic disciplines. Informed consent was obtained from school administrators, teachers, parents, and the students themselves prior to participation. The survey was conducted anonymously and confidentially, and student participation was entirely voluntary. This study was approved by the Ethics Committee of Huaibei Normal University (HBNU20250610). Questionnaires were distributed and collected online with the assistance of class instructors during scheduled sessions; this supervised, class-based administration accounts for the high response rate. A total of 984 questionnaires were returned, yielding a response rate of 98.4%. After excluding invalid responses based on criteria such as ‘completion of less than 85% of the items’, ‘obvious response patterns indicative of random answering’ and ‘regular or patterned responses’, 883 valid questionnaires were retained, resulting in an effective response rate of 89.7%. The final sample had a mean age of 19.15 years (SD = 1.08). The demographic characteristics of the participants were as follows: 389 males (44.1%) and 494 females (55.9%); 243 first-year students (27.5%), 256 second-year students (29.0%), 241 third-year students (27.3%), and 143 fourth-year students (16.2%); 583 students from humanities and social sciences backgrounds (66.0%) and 300 from natural sciences backgrounds (34.0%); 644 participants from urban areas (73.0%) and 239 from rural areas (27.0%); 285 only-child participants (32.3%) and 598 participants with siblings (67.7%). No *a priori* sample-size calculation was conducted; instead, a *post hoc* sensitivity analysis was performed to identify the smallest effect the design could reliably detect. With the final sample of 883 participants, a Type I error rate of 0.05, and statistical power of 0.80, the study was sufficiently powered to detect small effects, corresponding to a minimum detectable correlation of approximately r = 0.094 and, for individual regression coefficients in a model with up to five predictors, an effect size of f-squared of about 0.009. The achieved sample therefore provided adequate sensitivity for the small-to-medium associations hypothesized in the present chain-mediation model.

### 3.2 Measures

#### 3.2.1 Green exercise

This study employed the Intentional Nature Exposure Scale (INES) validated by Wood et al. (2019) to assess individuals’ participation in green exercise. The scale comprises five items spanning three domains of intentional nature engagement: nature contact in everyday life, nature contact outside everyday life, and nature exposure during physical activity (green exercise). Each item is rated on 1 to 5-point scale whose anchors denote either the frequency of engagement (for example, from once a weekly or less to year) or the amount of attention paid to nature (from not much to a great deal), and higher total scores indicate greater intentional engagement with green natural environments, including green exercise. The scale has undergone cross-cultural validation and has demonstrated good reliability and validity across different populations; in the present study, the scale demonstrated good internal consistency. The αcoefficient of internal consistency of the scale was 0.83.

#### 3.2.2 Mobile phone addiction

Mobile phone addiction was assessed using the Smartphone Addiction Scale for College Students, developed by Su Shuang and colleagues (Su et al., 2014). This scale has been widely employed in research investigating smartphone addiction among university students (Hu et al., 2021). The scale comprises 22 items across six dimensions: withdrawal behavior, salience, social comfort, negative consequences, application usage, and application updates. Items are rated on a 5-point Likert scale ranging from 1 ‘strongly disagree’ to 5 ‘strongly agree’. Higher total scores indicate more severe smartphone addiction. In the present study, the scale demonstrated good internal consistency. The *α* coefficient of internal consistency of the scale was 0.87.

#### 3.2.3 Perceived social support

Perceived social support was measured using the Perceived Social Support Scale (PSSS), as revised by Qj (2024). The scale consists of 12 items assessing perceived social support across three dimensions: family support (Items 3, 4, 8, and 11), friend support (Items 6, 7, 9, and 12), and significant other support (Items 1, 2, 5, and 10). Each item is rated on a 7-point Likert scale ranging from 1 (strongly disagree) to 7 (strongly agree), yielding total scores ranging from 12 to 84. Higher total scores indicate greater levels of perceived social support. The *α* coefficient of internal consistency of the scale was 0.95.

#### 3.2.4 Negative emotions

Negative emotions were assessed using the Chinese version of the 21-item Depression Anxiety Stress Scale (DASS-21), originally developed by Lovibond and Lovibond and revised by Gong et al. (2010). The DASS-21 comprises three dimensions depression, anxiety, and stress, each containing seven items, for a total of 21 items. Items are rated on a 4-point Likert scale ranging from 0 (did not apply to me at all) to 3 (applied to me very much, or most of the time). Higher total scores indicate more intense experiences of negative emotions. The α coefficient of internal consistency of the scale was 0.91.

#### 3.2.5 Test for multicollinearity

The VIF value was used to evaluate multicollinearity among the variables, with a value of > 10 representing high multicollinearity (Li et al., 2022). SPSS 23.0 was used to perform the multicollinearity test. Results revealed that the VIF value of all variables was less than 8, indicating that no multicollinearity was found among variables.

#### 3.2.6 Common method deviation test

To minimize the potential influence of common method bias on the study results, several procedural remedies were implemented prior to questionnaire administration. Participants received standardized training on the survey procedures, and the questionnaire employed clear instructional guidelines, strictly controlled completion times, incorporated a mix of positively and negatively worded items, and included controls for demographic variables. Following data collection, Harman’s single-factor test was conducted to statistically examine the presence of common method bias. All items pertaining to the study variables, including green exercise, perceived social support, negative emotions, and mobile phone addiction, were entered into an exploratory factor analysis. The results of the unrotated factor solution revealed five factors with eigenvalues greater than 1. The first factor accounted for 27% of the total variance, which falls below the recommended threshold of 40% (Tang and Wen, 2020). These findings indicate that common method bias was not a significant concern in the present study.

Because Harman’s single-factor test is now widely regarded as insufficient on its own, two additional procedures were applied. First, an unmeasured common latent factor (CLF) was added to the measurement model, and the standardized item loadings estimated with and without the CLF were compared; all differences were below 0.20, indicating that no single method factor accounted for a substantial share of the item variance. Second, a marker-variable test using a theoretically unrelated construct produced only negligible changes in the magnitude and significance of the focal correlations. Together with Harman’s test, these results suggest that common method bias was unlikely to have materially distorted the findings, although it cannot be entirely excluded given the single-source, single-occasion design.

### 3.3 Statistical analysis

Statistical analyses were conducted using SPSS version 23.0 and the PROCESS macro for SPSS developed by Hayes (2013). Prior to hypothesis testing, reliability and validity analyses, correlation analyses, and regression analyses were sequentially performed on the study variables to examine the psychometric properties and bivariate relationships among the measures. To test the mediating effects, the PROCESS macro was employed. Specifically, Model 4 of the PROCESS macro was used to examine the simple mediating effects of perceived social support and negative emotions individually, while Model 6 was utilized to test the chain mediating effect of perceived social support and negative emotions in sequence. All analyses employed bias-corrected bootstrap resampling methods to generate confidence intervals for the indirect effects, thereby enhancing the robustness of the mediation analyses.

## 4 Results

### 4.1 Descriptive statistics and correlation analysis

The results of the correlation analysis are presented in Table 1. As hypothesized, green exercise was significantly positively correlated with perceived social support (r = 0.336, *p* < 0.01) and significantly negatively correlated with both negative emotions (r = −0.376, *p* < 0.01) and mobile phone addiction (r = −0.475, *p* < 0.01). Perceived social support demonstrated significant negative correlations with negative emotions (r = −0.355, *p* < 0.01) and mobile phone addiction (r = −0.244, *p* < 0.01). Furthermore, negative emotions were significantly positively correlated with mobile phone addiction (r = 0.241, *p* < 0.01).

**Table 1:** The result of descriptive statistics and correlation analysis.

Variables	*M*	*SD*	1	2	3	4
1. Green exercise	18.321	4.341	1			
2. Perceived social support	66.242	11.347	0.336**	1		
3. Negative emotions	38.714	5.122	−0.376**	−0.355**	1	
4. Smart phone addiction	75.821	12.647	−0.475**	−0.244**	0.241**	1

*n* = 883; **p < 0.01; *p < 0.05. Table entries are Pearson correlation coefficients (r).

### 4.2 Mediating effects of perceived social support and negative emotions

The correlation analysis results satisfied the statistical prerequisites for further testing the mediating effects of perceived social support and negative emotions (Wen and Ye, 2014). Subsequently, with gender, age, academic major, and only-child status included as control variables, mediation analyses were conducted using the PROCESS macro for SPSS (Model 6) developed by Hayes (2013), employing bootstrap resampling methods to test the chain mediating effect.

As shown in Table 2, green exercise was significantly and negatively associated with mobile phone addiction among university students (*β* = −0.214, *p* < 0.01), supporting Hypothesis 1. After incorporating perceived social support and negative emotions into the regression equation, green exercise was significantly and positively associated with perceived social support (*β* = 0.645, *p* < 0.01) and significantly and negatively associated with negative emotions (*β* = −0.326, *p* < 0.01); perceived social support was significantly and negatively associated with both negative emotions (*β* = −0.314, *p* < 0.01) and mobile phone addiction (*β* = −0.144, *p* < 0.01); and negative emotions were significantly and positively associated with mobile phone addiction (*β* = 0.278, *p* < 0.01). The total effect of green exercise on mobile phone addiction was significant (*β* = −0.454, *p* < 0.01); after perceived social support and negative emotions were entered as mediators, the direct effect was reduced in magnitude but remained significant (*β* = −0.214, *p* < 0.01), indicating partial mediation.

**Table 2:** Analysis of regression relationship among variables.

Effect	Term	Effect	*SE*	*t*	*p*	LLCI	ULCI
Direct effect	Green exercise ⇒ Smart phone addiction	−0.214	0.032	−6.688	<0.001	−0.277	−0.151
Path coefficients	Green exercise ⇒ Perceived social support	0.645	0.021	30.714	<0.001	0.604	0.686
	Green exercise ⇒ Negative emotions	−0.326	0.029	−11.241	<0.001	−0.383	−0.269
	Perceived social support ⇒ Negative emotions	−0.314	0.024	−13.083	<0.001	−0.361	−0.267
	Perceived social support ⇒ Smart phone addiction	−0.144	0.023	−6.261	<0.001	−0.189	−0.099
	Negative emotions ⇒ Smart phone addiction	0.278	0.032	8.688	<0.001	0.215	0.341
Total effect	Green exercise ⇒ Smart phone addiction	−0.454	0.030	−15.133	<0.001	−0.513	−0.395

LLCI refers to the lower limit of the 95% interval of the estimated value, while ULCI refers to the upper limit of the 95% interval of the estimated value.

**Figure 2:**
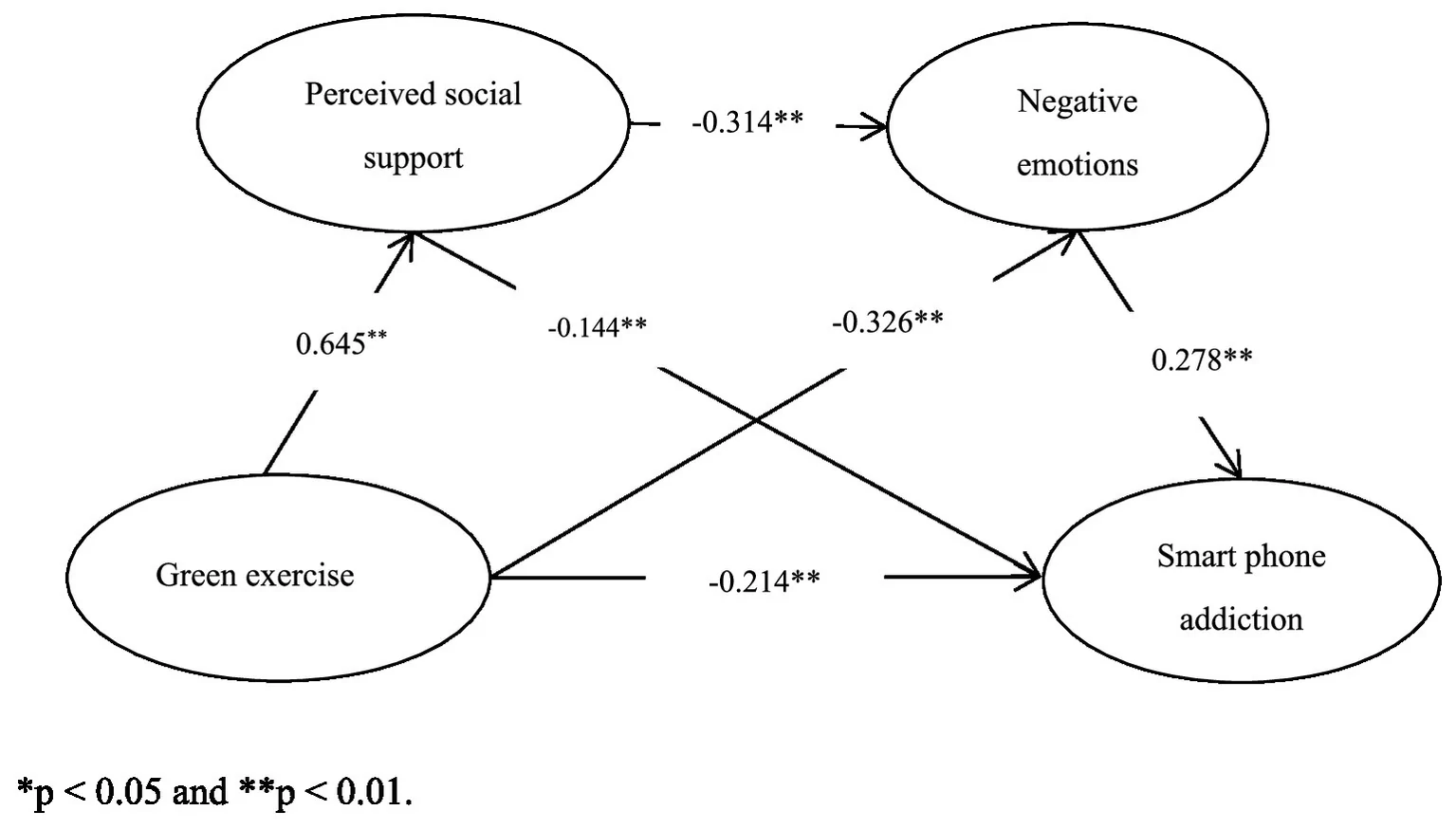
The chain mediation model of perceived social support and negative emotions.

The results of the mediation effect analysis are presented in Table 3 and Figure 2. Using bootstrap resampling with 5,000 samples, the indirect effects were examined. For the mediation pathway ‘green exercise ⇒ perceived social support⇒ mobile phone addiction’, the 95% confidence interval did not contain 0(95% CI: [−0.122, −0.064]), indicating a significant indirect effect. Similarly, for the pathway ‘green exercise⇒ negative emotions⇒ mobile phone addiction’, the 95% confidence interval excluded 0 (95% CI: [−0.116, −0.066]), confirming the presence of this mediating effect. Furthermore, the chain mediation pathway ‘green exercise⇒ perceived social support⇒ negative emotions⇒ mobile phone addiction’ also yielded a 95% confidence interval that did not include 0 (95% CI: [−0.072, −0.040]), demonstrating a significant chain mediating effect. Accordingly, Hypotheses 2, 3, and 4 were all supported.

**Table 3:** Mediating effect analysis of perceived social support and negative emotions.

Term	Effect	Boot SE	BootLLCI	BootULCI	*z*	*p*
Green exercise ⇒ Perceived social support ⇒ Smart phone addiction	−0.093	0.015	−0.122	−0.064	−6.20	<0.001
Green exercise ⇒ Negative emotions ⇒ Smart phone addiction	−0.091	0.013	−0.116	−0.066	−7.00	<0.001
Green exercise ⇒ Perceived social support ⇒ Negative emotions ⇒ Smart phone addiction	−0.056	0.008	−0.072	−0.040	−7.00	<0.001
Total indirect effect	−0.240	0.022	−0.283	−0.197	−10.91	<0.001

*p < 0.05 and **p < 0.01.

## 5 Discussion

### 5.1 Green exercise and mobile phone addiction

The present study found that green exercise was negatively associated with mobile phone addiction among university students, and this association remained statistically significant after perceived social support and negative emotions were taken into account. This pattern is consistent with prior work showing that physical activity and problematic technology use tend to vary inversely: heavier mobile phone use has been associated with lower physical activity among Chinese students (Han et al., 2023; Feng et al., 2022) and, in a moderated-mediation study of Tunisian university students, higher physical activity was associated with lower problematic internet use (Jelleli et al., 2024). Several accounts may help explain why green exercise is linked to lower mobile phone addiction, although the present data cannot adjudicate among them. As an offline health behavior, green exercise occupies time and attention that might otherwise be devoted to the phone, a displacement process suggested by earlier studies of exercise and screen use in students (Yang, 2019; Dong et al., 2023); in addition, the restorative experience of natural settings may support self-regulation and reduce reliance on the phone as a source of gratification. Because these processes were not measured, they are offered as interpretations of the observed association rather than as tested mechanisms.

### 5.2 The mediating role of perceived social support

Green exercise was positively associated with perceived social support, which was in turn negatively associated with mobile phone addiction, indicating an indirect association operating through perceived social support. Group-based forms of green exercise, such as outdoor hiking or collective fitness activities, provide recurrent opportunities for interpersonal contact and mutual assistance, which may strengthen students’ subjective sense of being supported (Bai, 2022). Consistent with a social-buffering perspective, students who perceive that support is available in their real-world relationships may be less inclined to use the smartphone to compensate for unmet relational needs or to escape interpersonal difficulties, and may appraise their own phone use more adaptively (Zhang et al., 2021; Wang and Yao, 2025). These interpretations are compatible with the observed association but were not tested directly in the present cross-sectional data.

### 5.3 The mediating role of negative emotions

Green exercise was negatively associated with negative emotions, which were in turn positively associated with mobile phone addiction, indicating a second indirect association operating through affect. This affective pathway parallels the findings of Jelleli et al. (2024), who reported that the association between physical activity and problematic internet use was partly accounted for by depression, anxiety, and stress assessed with the DASS-21, the same instrument used in the present study. Two complementary interpretations are frequently advanced in this literature but were not evaluated here. On the one hand, smartphones offer immediate and low-cost emotional relief, so their repeated use during negative affective states may become a habitual coping strategy that further burdens self-regulatory resources (Zhou et al., 2017; Tong et al., 2019). On the other hand, physical activity in natural environments has been linked to lower physiological stress and improved mood; to the extent that green exercise is accompanied by lower negative affect, the emotional impetus to over-use the phone may be attenuated. As these mechanisms were not measured, they should be read as plausible interpretations rather than as established processes.

### 5.4 The chain association of perceived social support and negative emotions

Finally, the data were consistent with a chain association in which green exercise related to higher perceived social support, higher perceived social support related to lower negative emotions, and lower negative emotions related to lower mobile phone addiction. This sequence integrates the social-cognitive and affective pathways: perceived support may not only reduce direct reliance on the phone but also buffer negative affect, which is itself associated with problematic use. It should be emphasized that this ordering is only one of several statistically equivalent accounts of the same associations; because the design is cross-sectional, the temporal order among the variables cannot be confirmed, and reverse or reciprocal relations remain plausible (see Limitations). The findings are therefore best understood as identifying a coherent set of correlates that may help explain the association between green exercise and lower mobile phone addiction, rather than as evidence of a causal chain or of an intervention effect.

## 6 Limitations

The present study examined the combined effects of green exercise, perceived social support, and negative emotions on mobile phone addiction among university students, yielding findings that offer practically relevant insights for the development of prevention strategies targeting smartphone addiction in this population. Nevertheless, several limitations should be acknowledged. First, and most important, the design is cross-sectional; the data therefore describe associations at a single point in time and cannot establish temporal precedence or causal direction. The hypothesized ordering (from green exercise to perceived social support, to negative emotions, and to mobile phone addiction) is only one of several statistically equivalent representations of the same set of covariances, and reverse or reciprocal relations cannot be ruled out; indeed, heavier mobile phone addiction has itself been reported to relate to lower physical activity (Feng et al., 2022). All results reported here should therefore be read as associations rather than as evidence of influence or of an intervention benefit. Second, all four constructs were assessed by self-report from a single source on a single occasion, so common method variance is a concern; although Harman’s single-factor test, an unmeasured common latent factor, and a marker variable each indicated that method bias was unlikely to be severe, it cannot be entirely excluded. Third, although a stratified cluster framework was used, the questionnaires were administered online in class-based sessions, a procedure that may limit the representativeness of the sample and introduce coverage and self-selection bias; non-response was not examined in detail. Fourth, mobile phone addiction is multidimensional and is associated with additional factors that were not modeled here, such as impulsivity and implicit cognitions.

Future research should test the proposed ordering using longitudinal or experimental designs, recruit participants through procedures that better support representativeness, and complement self-report with objective measures. For digital behavior in particular, objective indices of screen exposure, together with device-based monitoring of physical activity, would reduce shared-method bias and allow the associations reported here to be tested more rigorously.

## 7 Conclusion and future directions

In this cross-sectional sample of university students, green exercise was negatively associated with mobile phone addiction, both directly and indirectly through perceived social support and negative emotions, including their sequential (chain) pathway. Specifically, greater engagement in green exercise was associated with greater perceived social support, which was in turn associated with lower negative emotions and, ultimately, with a lower level of mobile phone addiction. These findings identify social-cognitive and emotional correlates that may help account for the association between green exercise and problematic smartphone use in this population.

## Data Availability

The data that support the findings of this study are available from the corresponding author upon reasonable request.
